# Reported use of reporting guidelines among *JNCI: Journal of the National Cancer Institute* authors, editorial outcomes, and reviewer ratings related to adherence to guidelines and clarity of presentation

**DOI:** 10.1186/s41073-018-0052-4

**Published:** 2018-09-27

**Authors:** Jeannine Botos

**Affiliations:** Oxford University Press, 198 Madison Ave, New York, NY 10016 USA

**Keywords:** Submissions, Editorial decisions, Clarity, Presentation, Reporting guidelines, Adherence, Peer review

## Abstract

**Background:**

Associations were examined between author-reported uses of reporting guidelines to prepare *JNCI: Journal of the National Cancer Institute* (*JNCI*) submissions, editorial decisions, and reviewer ratings for adherence to reporting guidelines and clarity of presentation.

**Methods:**

At submission, authors were asked if they used reporting guidelines to prepare their manuscript and, if so, which one(s). Reviewers rated adherence to reporting guidelines and clarity of presentation. Data were gathered using a customized Editorial Manager Enterprise Analytics Report for submissions with first or final decisions that were submitted between November 1, 2015, and April 30, 2017. Manuscript types that would benefit from the use of reporting guidelines were included. All reviews were included in the analyses. Numerical values were given to each answer (yes, 1; no, 0) or reviewer rating (not applicable, 0; fair, 1; poor, 2; good, 3; very good, 4; and outstanding, 5), and scores were compared using two-sided *t* tests.

**Results:**

Of 2209 submissions included in the analysis, 1144 (51.8%) indicated that at least one reporting guideline was used. The STROBE guidelines were the most common (*n* = 531, 24.0%). Of the 2068 (93.6%) submissions that were rejected, 1105 (50.1%) indicated using reporting guidelines and 963 (43.6%) did not (mean [SD] scores of rejected vs not rejected, 0.53 [0.50] vs 0.49 [0.50], *P* = .47). Of the 1033 ratings for adherence to reporting guidelines, mean (SD) scores for not rejected vs rejected submissions were 3.2 (1.61) vs 2.9 (1.57) (*P* = .005), and mean (SD) scores for reporting guidelines use vs no use were 3.1 (1.48) vs 2.9 (1.70) (*P* = .01). Of the 1036 ratings for clarity of presentation, mean (SD) scores for not rejected vs rejected submissions were 3.6 (1.00) vs 3.1 (1.08) (*P* < .001), whereas mean (SD) scores for reporting guidelines use vs no use were 3.3 (1.04) vs 3.3 (1.10) (*P* = .64).

**Conclusions:**

Among these *JNCI* submissions, reporting the use of reporting guidelines was not associated with editorial decisions or with reviewer ratings for clarity of presentation. Reviewer ratings for adherence to guidelines and clarity of presentation were associated with editorial decisions after peer review, and ratings for adherence to guidelines were associated with reported use of reporting guidelines.

## Background

To date, approximately 400 reporting guidelines currently exist [1]; all were designed to improve the quality of reporting in the published literature [2]. Although one way to adhere to the guidelines is to incorporate the points in the guidelines before final publication, using these guidelines to prepare manuscripts could ensure complete reporting of methods and results and improve the clarity of the presentation; thus, these manuscripts would be easier to understand and critique by peer reviewers and editors. However, to my knowledge, there has not previously been such a study to prove this.

With the demand for publishing being high and with many journals receiving substantially more manuscripts than they can publish and turning many away rapidly, editorial offices may not want to put additional burden on authors beyond the policies, formatting, and word restrictions they already impose on authors at submission. However, editors know the importance of the reporting guidelines [3, 4] and find themselves in a dilemma. Some journals require that reporting guidelines checklists be submitted along with the files at initial submission; other journals do not.

The *JNCI: Journal of the National Cancer Institute* (*JNCI*) publishes a broad range of study designs with outcomes related to cancer. It uses a multi-tiered editorial process to triage submissions for further review by expert editors and peer and statistical reviewers. Its acceptance rate is in the single digits, with greater than 75% of submissions being rejected before peer review. *JNCI* has enforced the use of the CONsolidated Standards of Reporting Trials (CONSORT) checklist at submission for clinical trials for many years [5], but other reporting guidelines are not enforced, although they are all strongly encouraged in the Journal’s Author Guidelines. Reporting standards are enforced before publication.

The main goals of this study were to find out if associations exist between authors claiming to use reporting guidelines to prepare their submissions, editorial outcomes, and reviewer ratings related to adherence to reporting guidelines and clarity of presentation. The results may help editors decide whether requiring reporting guidelines at submission is necessary and worth the effort.

## Methods

### Authors and reviewers were surveyed

At submission, authors were asked if they used any of the following reporting guidelines to prepare their manuscript and, if so, which one(s): ARRIVE [6] for animal studies; REMARK [7] for prognostic markers; STARD [8] for diagnostic markers; MOOSE [9] for meta-analyses of observational studies in medicine; PRISMA [10] for systematic reviews and meta-analyses of health care interventions; STROBE [11] for cohort and case-control studies; STREGA—STROBE Extension to Genetic Association studies: STrengthening the REporting of Genetic Association studies [12]; MIQE [13] for studies that use qPCR experiments; BRISQ—Biospecimen Reporting for Improved Study Quality, particularly Tier 1 items, for studies using biospecimens [14]; CONSORT [15] for randomized clinical trials; Other, with a space to enter the reporting guideline(s) used; and Not Applicable. *JNCI*’s review process requires reviewers to subjectively rate adherence to reporting guidelines and clarity of presentation on a form using a scale (not applicable, fair, poor, good, very good, and outstanding) as part of their critique.

### Data collection

Data were gathered using a customized Editorial Manager Enterprise Analytics Report. Submissions with first or final decisions that were submitted between November 1, 2015, and April 30, 2017, were included in the study. This period was chosen because editorial decision-making processes were consistent. Manuscript types that would benefit from the use of reporting guidelines were included, i.e., Articles, Brief Communications, Reviews, Meta-analyses, and Systematic Reviews. Manuscript types that did not have a reporting guideline applicable were excluded, i.e., Commentaries, Editorials, Correspondence, and Response. There were no exclusions otherwise. Numerical values were given to each answer (yes, 1; no, 0).

Each submission received 1–3 peer reviews and 1 statistical review. All completed reviews were included in the analysis. Numerical values were given to each reviewer rating (not applicable, 0; fair, 1; poor, 2; good, 3; very good, 4; and outstanding, 5).

The data were normally distributed by visual inspection. Therefore, means (SD) were calculated and compared using two-sided *t* tests using Microsoft Excel. *P* < .05 was considered statistically significant, and no adjustment for multiple comparisons was made.

## Results

### General metrics of submissions included in the study

A total of 2209 submissions were included in the analysis. Of these, 1715 (77.6%) were priority rejected, 98 (4.4%) were rejected without review, and 396 (17.9%) were peer reviewed. Of the 396 that were peer reviewed, 255 (11.5% of the total) were rejected and 141 (6.4% of the total) were not rejected.

### Uptake of reporting guidelines

**Fig. 1:**
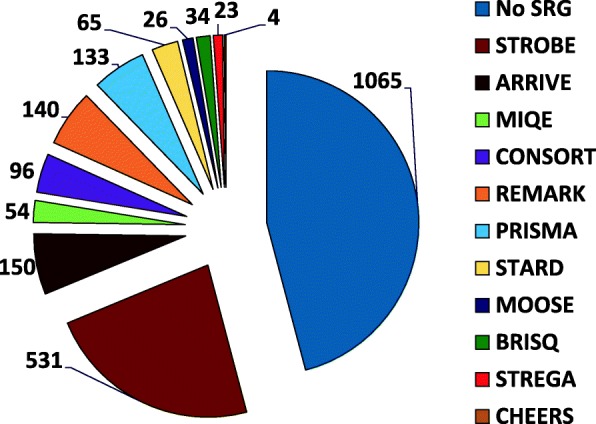
Numbers of submissions for which authors said they used a reporting guideline or did not. *Standard reporting guideline* (*SRG*); *Strengthening-Reporting of Observational-Studies in Epidemiology* (STROBE) [11]; *Animal Research: Reporting* In Vivo *Experiments (*ARRIVE) [6]; Minimum Information for Publication of Quantitative Real-Time PCR Experiments (MIQE) [13]; Consolidated Standards of Reporting Trials (CONSORT) [15]; REporting recommendations for tumour MARKer prognostic studies (REMARK) [7]; Preferred Reporting Items for Systematic Reviews and Meta-analyses (PRISMA) [10]; studies of diagnostic accuracy (STARD) [8]; Meta-analyses of Observational Studies (MOOSE) [9]; Biospecimen reporting for improved study quality (BRISQ) [14]; STrengthening the REporting of Genetic Association Studies (STREGA) [12], an extension to STROBE; and Consolidated Health Economic Evaluation Reporting Standards (CHEERS) [16]

First, the uptake of standard reporting guidelines was calculated for the study period (Fig. 1, Table 1). Of 2209 submissions included in the analysis, 1144 (51.8%) indicated that at least one reporting guideline was used. The STROBE guidelines were the most common (*n* = 531, 24.0%).

**Table 1:** Submissions by editorial decision and by the reporting guidelines authors said they used

Editorial decision	All Submissions	STROBE	ARRIVE	MIQE	CONSORT	REMARK	PRISMA	STARD	MOOSE	BRISQ	STREGA	CHEERS
All, no. (%)	2209 (100)	531 (24.0)	150 (6.8)	54 (2.4)	96 (4.3)	140 (6.3)	133 (6.0)	65 (2.9)	26 (1.2)	34 (1.5)	23 (1.0)	4 (0.2)
Sent to peer review	396 (17.9)	86 (3.9)	28 (1.3)	5 (0.2)	23 (1.0)	24 (1.1)	25 (1.1)	10 (0.5)	7 (0.3)	4 (0.2)	7 (0.3)	0 (0.0)
Rejected after peer review	255 (11.5)	36 (1.6)	14 (0.6)	3 (0.1)	7 (0.3)	12 (0.5)	16 (0.7)	5 (0.2)	1 (0.0)	3 (0.1)	4 (0.2)	0 (0.0)
Not rejected after peer review	141 (6.4)	50 (2.3)	14 (0.6)	2 (0.1)	16 (0.7)	12 (0.5)	9 (0.4)	5 (0.2)	6 (0.3)	1 (0.0)	3 (0.1)	0 (0.0)
Not sent to peer review	1813 (82.1)	445 (20.1)	122 (5.5)	49 (2.2)	73 (3.3)	116 (5.3)	108 (4.9)	55 (2.5)	19 (0.9)	30 (1.4)	16 (0.7)	4 (0.2)
Reject without review	98 (4.4)	22 (1.0)	1 (0.0)	1 (0.0)	2 (0.1)	2 (0.1)	8 (0.4)	2 (0.1)	1 (0.0)	2 (0.1)	0 (0.0)	2 (0.1)
Priority reject	1715 (77.6)	423 (19.1)	121 (5.5)	48 (2.2)	71 (3.2)	114 (5.2)	100 (4.5)	53 (2.4)	18 (0.8)	28 (1.3)	16 (0.7)	2 (0.1)

*STROBE* Strengthening-Reporting of Observational-Studies in Epidemiology, *ARRIVE* Animal Research: Reporting In Vivo Experiments, *MIQE* Minimum Information for Publication of Quantitative Real-Time PCR Experiments, *CONSORT* Consolidated Standards of Reporting Trials, *REMARK* Reporting recommendations for tumour Marker prognostic studies, *PRISMA* Preferred Reporting Items for Systematic Reviews and Meta-analyses, *STARD* studies of diagnostic accuracy, *MOOSE* Meta-analyses of Observational Studies, *BRISQ* Biospecimen reporting for improved study quality, *STREGA* Strengthening the Reporting of Genetic Association Studies , an extension to STROBE; and Consolidated Health Economic Evaluation Reporting Standards (CHEERS)

### Relationship between the author’s claim of using reporting guidelines and editorial decisions

The next question was whether the author’s claim of using reporting guidelines was associated with editorial decisions. Of the 2068 (93.6%) submissions that were rejected, 1105 (50.1%) indicated using reporting guidelines and 963 (43.6%) did not (mean [SD] scores of rejected vs not rejected, 0.53 [0.50] vs 0.49 [0.50], *P* = .47, Table 2). Therefore, there was no relationship between authors claiming to use reporting guidelines and editorial decisions.

**Table 2:** Use of reporting guidelines scores across editorial decisions

Editorial decision	Mean score^a^ (SD)	*P*
Rejected without peer review	0.53 (0.50)	
Rejected after peer review	0.53 (0.50)	.68
Not rejected after peer review	0.49 (0.50)	.47

^a^Submissions were scored according to the following rules: authors indicated they used a reporting guideline to prepare their submission, 1; authors indicated they did not use a reporting guideline to prepare their submission, 0. *P* values were calculated using a two-sided paired *t* test

### Relationship between reviewer ratings and editorial decisions

The next question was whether reviewer ratings were associated with editorial decisions (Table 3). Of the 1033 ratings for adherence to reporting guidelines, mean (SD) scores for not rejected vs rejected submissions were 3.2 (1.61) vs 2.9 (1.57) (*P* = .005). Of the 1036 ratings for clarity of presentation, mean (SD) scores for not rejected vs rejected submissions were 3.6 (1.00) vs 3.1 (1.08) (*P* < .001). Therefore, there was a direct association between reviewer ratings and editorial decisions.

**Table 3:** Reviewer rating scores across editorial decisions^a^

Editorial decision	Adherence to reporting guidelines		Clarity of presentation	
	Reviewer rating score, mean (SD)	*P*	Reviewer rating score, mean (SD)	*P*
Rejected after peer review	2.9 (1.57)		3.1 (1.08)	
Not rejected after peer review	3.2 (1.61)	.005	3.6 (1.00)	< .001

^a^Reviewer rating, score: not applicable, 0; fair, 1; poor, 2; good, 3; very good, 4; and outstanding, 5. *P* values were calculated using a two-sided paired *t* test

### Relationship between author’s claim of using reporting guidelines and reviewer ratings

The final question was whether there was a relationship between author’s claim of using reporting guidelines and reviewer ratings (Table 4). For adherence to reporting guidelines, mean (SD) scores for claiming to use reporting guidelines vs no use were 3.1 (1.48) vs 2.9 (1.70) (*P* = .01). For clarity of presentation, mean (SD) scores for reporting guidelines use vs no use were 3.3 (1.04) vs 3.3 (1.10) (*P* = .64). In this case, author’s claim of use of reporting guidelines was associated with reviewer ratings for adherence to guidelines, but not with reviewer ratings for clarity of presentation.

**Table 4:** Reviewer rating scores according to author’s claim of using reporting guidelines

Reviewer question	Author said they used a reporting guideline to prepare their submission		
	No	Yes	*P*
Adherence to reporting guidelines	2.9 (1.70)	3.1 (1.48)	.01
Clarity of presentation	3.3 (1.10)	3.3 (1.04)	.64

Authors reported using the following reporting guidelines: *STROBE* Strengthening-Reporting of Observational-Studies in Epidemiology, *ARRIVE* Animal Research: Reporting In Vivo Experiments, *MIQE* Minimum Information for Publication of Quantitative Real-Time PCR Experiments, *CONSORT* Consolidated Standards of Reporting Trials, *REMARK* Reporting recommendations for tumour Marker prognostic studies, *PRISMA* Preferred Reporting Items for Systematic Reviews and Meta-analyses, *STARD* Studies of diagnostic accuracy, *MOOSE* Meta-analyses of Observational Studies, *BRISQ* Biospecimen reporting for improved study quality, *STREGA* STrengthening the REporting of Genetic Association Studies, an extension to STROBE; and *CHEERS* Consolidated Health Economic Evaluation Reporting Standards. Some percentages do not add to 100 owing to rounding. Numerical values were given to each answer (SRG use, 1; no SRG use, 0) or reviewer rating (not applicable, 0; fair, 1; poor, 2; good, 3; very good, 4; and outstanding, 5), and mean scores are presented. *P* values were calculated using a two-sided paired *t* test

## Discussion

In this study, author submission questionnaires, reviewer critiques, and editorial outcomes were analyzed to determine whether associations exist between authors claiming to use reporting guidelines to prepare their submissions, reviewer perceptions of clarity of reporting and adherence to reporting guidelines, and editorial decisions. A positive association between authors claiming to use reporting guidelines and reviewer ratings of adherence to reporting guidelines was found. Positive associations between reviewer ratings of adherence to reporting guidelines and editorial outcomes and of clarity of presentation and editorial outcomes were also observed, e.g., submissions with lower adherence to reporting guidelines scores and those with lower clarity of presentation scores more frequently received rejection decisions; those with higher ratings were more often accepted. However, there was no association between authors claiming to use reporting guidelines and reviewer perception of clarity of presentation. Also, there was no association between authors claiming to use reporting guidelines and editorial decisions, e.g., submissions on which authors claimed to use reporting guidelines were not more often accepted.

As with most studies, there are limitations. The main limitation is that the analysis was based in part on a survey of authors. Whether or not authors actually used the reporting guidelines to prepare their submissions was not independently confirmed. Another limitation is that it analyzed submissions to a single journal.

Although a limitation of the study is the survey methodology, the positive association between authors claiming to use reporting guidelines and reviewer ratings of adherence to reporting guidelines was reassuring, especially because reviewers could not see the author questionnaire on which this was recorded.

The lack of an association between the authors claiming to use reporting guidelines and reviewer ratings of clarity of presentation was surprising. One could assume that using reporting guidelines to prepare a submission would ensure transparency in reporting methodology and outcomes. This in turn should make the manuscript easier for reviewers to assess.

Unfortunately, it was not possible in this study to assess the editor’s perspective of adherence to guidelines and the clarity of the presentation, which might be helpful if a change in policy or process of enforcement is being considered.

Also, because the type of reporting guideline authors claimed to use varied widely across the study, it was not possible to compare the different guidelines. And, because relatively few trials were submitted, the submissions that used the CONSORT checklist could not be used as a comparison group.

## Conclusions

Submissions that were judged by reviewers as being more clearly presented were accepted for publication more often. Claiming to use a reporting guideline did not make a submission more clearly presented, according to reviewers, nor were these submissions more likely to be accepted. There was good concordance between authors claiming to use reporting guidelines and reviewer assessments of their use.

These results should not discourage authors from using reporting guidelines to prepare their manuscripts or discourage journals from enforcing them before publication, but they may help editors and editorial staff decide when and how to enforce them.

## Abbreviations

ARRIVE: Animal Research: Reporting In Vivo Experiments
BRISQ: Biospecimen reporting for improved study quality
CHEERS: Consolidated Health Economic Evaluation Reporting Standards
CONSORT: Consolidated Standards of Reporting Trials
JNCI: Journal of the National Cancer Institute
MIQE: Minimum Information for Publication of Quantitative Real-Time PCR experiments
MOOSE: Meta-analyses of Observational Studies
PRISMA: Preferred Reporting Items for Systematic Reviews and Meta-analyses
REMARK: Reporting recommendations for tumour marker prognostic studies
STARD: Studies of diagnostic accuracy
STREGA: Strengthening the Reporting of Genetic Association Studies, an extension to STROBE
STROBE: Strengthening-Reporting of Observational-Studies in Epidemiology

## References

[1] The EQUATOR Network: Enhancing the quality and transparency of health research. http://www.equator-network.org/. Accessed 16 Feb 2018.

[2] Wang X, Chen Y, Yang N, Deng W, Wang Q, Li N, Yao L, Wei D, Chen G, Yang K (2015). Methodology and reporting quality of reporting guidelines: systematic review. BMC Med Res Methodol.

[3] Grindlay DJC, Dean RS, Christopher MM, Brennan ML (2014). A survey of the awareness, knowledge, policies and views of veterinary journal Editors-In-Chief on reporting guidelines for publication of research. BMC Vet Res.

[4] Simera I, Moher D, Hirst A, Hoey J, Schulz KF, Altman DG. Transparent and accurate reporting increases reliability, utility, and impact of your research: reporting guidelines and the EQUATOR Network. BMC Med. 2010;8:24. 10.1186/1741-7015-8-24. Accessed 9 Aug 2018. 10.1186/1741-7015-8-24 PMC2874506 20420659

[5] Hopewell S, Altman DG, Moher D, Schulz KF (2008). Endorsement of the CONSORT Statement by high impact factor medical journals: a survey of journal editors and journal “Instructions to Authors”. Trials.

[6] Kilkenny C, Browne WJ, Cuthill IC, Emerson M, Altman D. Improving bioscience research reporting: the ARRIVE guidelines for reporting animal research. PLoS Biol. 2010. 10.1371/journal.pbio.1000412. 10.1371/journal.pbio.1000412 PMC2893951 20613859

[7] Sauerbrei W, Taube SE, McShane LM, Cavenaugh MM, Altman DG. Reporting recommendations for tumor marker prognostic studies (remark): an abridged explanation and elaboration. J Natl Cancer Inst. 2018:110(8):803–11. 10.1093/jnci/djy088. 10.1093/jnci/djy088 PMC6093349 29873743

[8] Bossuyt PM, Reitsma JB, Bruns DE, Gatsonis CA, Glasziou PP (2015). STARD 2015: an updated list of essential items for reporting diagnostic accuracy studies. BMJ.

[9] Stroup DF, Berlin JA, Morton SC, Olkin I, Williamson GD (2000). Meta-analysis of observational studies in epidemiology: a proposal for reporting. JAMA.

[10] Moher D, Liberati A, Tetzlaff J, Altman DG. The PRISMA group preferred reporting items for systematic reviews and meta-analyses: The PRISMA statement. PLoS Med. 6(7):e1000097. 10.1371/journal.pmed.1000097. 10.1371/journal.pmed.1000097 PMC2707599 19621072

[11] von Elm E, Altman DG, Egger M, Pocock SJ, Gøtzsche PC, Vandenbroucke JP, Initiative STROBE (2007). The Strengthening the Reporting of Observational Studies in Epidemiology (STROBE) statement: guidelines for reporting observational studies. Ann Intern Med.

[12] Little J, Higgins J, Ioannidis JPA, Moher D, Gagnon F (2009). STrengthening the REporting of Genetic Association studies (STREGA). PLoS Med.

[13] MIQE checklist for authors, reviewers, and editors. http://www.rdml.org/miqe.php. Accessed 16 Feb 2018.

[14] Moore HM, Kelly AB, Jewell SD, McShane LM, Clark DP, Greenspan R, Hayes DF, Hainaut P, Kim P, Mansfield EA, Potapova O, Riegman P, Rubinstein Y, Seijo E, Somiari S, Watson P, Weier H-U, Zhu C, Vaught J (2011). Biospecimen reporting for improved study quality (BRISQ). Cancer Cytopathol.

[15] CONSORT: Transparent reporting of Trials. http://www.consort-statement.org/. Accessed 16 Feb 2018.

[16] Husereau D, Drummond M, Petrou S, Carswell C, Moher D, Greenberg D, Augustovski F, Briggs AH, Mauskopf J, Loder E, on behalf of the CHEERS Task Force (2013). Consolidated Health Economic Evaluation. Reporting Standards (CHEERS) statement. J Med Econ.

